# UC-II® undenatured type II collagen data show retention during functional food and beverage prototype processing

**DOI:** 10.1016/j.dib.2023.109216

**Published:** 2023-05-09

**Authors:** George Pates, Tyler White, Shane Durkee, Zainulabedin Saiyed

**Affiliations:** a Quality Control, Lonza Greenwood LLC., 535 North Emerald Road, Greenwood, SC, USA; b Research and Development, Lonza Greenwood LLC., 535 North Emerald Road, Greenwood, SC, USA

**Keywords:** Undenatured type II collagen, Joint health, Functional nutrients, Foods, Beverages

## Abstract

Nowadays, collagen is widely used in food and beverage industries to enhance the nutritional and health value of the products. While many see this as an ideal way to incorporate more collagen into their diets, the exposure of these proteins to high temperature or acidic and alkaline solutions may negatively affect the quality and activity of these supplements. In general, the manufacturing of functional food and beverages often largely depends on the stability of the active ingredients during processing. The high temperatures, humidity, and low pH of processing may reduce product nutrient retention. Hence, understanding stability of collagen is of great significance and these data were gathered to determine the extent of undenatured type II collagen retention under different processing conditions. UC-II® undenatured type II collagen is a patented form of collagen derived from chicken sternum cartilage, and different food and beverage prototypes incorporating UC-II® undenatured type II collagen were produced. The content of undenatured type II collagen was compared in their pre-and post-manufacturing formats using an enzyme-linked immunosorbent assay. The undenatured type II collagen retention varied depending upon the prototype, with the highest amount of undenatured type II collagen retention occurring in nutritional bars (approximately 100%), followed by chews (98%), gummies (96%), and dairy beverages (81%). The present work also showed that recovery of the undenatured type II collagen depends on the exposure time, temperature and pH of the prototype.

**Specifications Table**

**Table:** 

Subject	Food Science: Food Chemistry
Specific subject area	The stability of undenatured type II collagen through the manufacturing processes of functional foods and beverages
Type of data	Table Figure
How the data were acquired	Solids collected from food and beverage samples containing UC-II® undenatured type II collagen were evaluated using an enzyme-linked immunosorbent assay (ELISA) before and after undergoing a variety of processing protocols. A multispecies Type II Collagen Detection Kit from Chondrex, Inc (Woodinville, WA, USA; Harris et al., 2021) was used in performing the ELISAs.
Data format	Raw Analyzed
Description of data collection	UC-II®-containing functional food and beverage prototypes were selected to represent a variety of manufacturing processes. These prototypes included nutritional bars, gummies, chews, dairy milk, chocolate milk, plant-based beverages, and carbonated beverages. The amount (in mg) of undenatured type II collagen was detected via ELISA for each raw, pre-processed prototype and again for its post-processing product.
Data source location	Lonza Greenwood LLC Greenwood, SC USA Latitude: 34.2089355 Longitude: −82.1221884
Data accessibility	https://data.mendeley.com/datasets/wxsddh5by3

## Value of the Data

• The retention of UC-II® undenatured type II collagen through various processes is a prerequisite for product development and must be supported by appropriate stability studies.
• Manufacturers can use the data to support the selection of UC-II® undenatured type II collagen as an ingredient with stability adequate for functional food and beverage product processing.
• Determine ideal conditions for maximum UC-II® undenatured type II collagen retention as comparators for other manufacturing processes.
• The retention of active UC-II® undenatured type II collagen in functional food and beverage products has the potential to optimize health and wellness.

## 1 Objective

Functional ingredients in food and beverage products can be affected by various conditions during the manufacturing and extrusion processes including temperature, humidity, and low pH [1,2]. Moreover, following processing, protein is particularly susceptible to alteration in its digestibility and bioavailability [3]. In extrusion processing, thermal degradation is the major factor contributing to denaturation of collagen [4]. Therefore, these data were collected to determine the extent of UC-II retention under different processing conditions for various functional food and beverage products.

## 2 Data Description

Tables 2 through 4 present the pre-and post-processing levels of undenatured type II collagen in tested prototypes. The nutritional bars showed the highest retention of undenatured type II collagen, followed by the other products (Table 2). Nine of the prototypes had *a* > 50% retention of undenatured type II collagen. More than 95% of the undenatured type II collagen was recovered in the gummies and chews. There was some loss of undenatured type II collagen in the brown rice chips, which were exposed to high temperature. The retention of undenatured type II collagen also varied by time of exposure to the food matrix based on the processing condition.

Table 3 reports the retention of undenatured type II collagen in dairy beverages. Although pasteurization, homogenization, and ultra-high treatment (UHT) processes, including exposure time, may reduce the level of retained undenatured type II collagen, the UHT processed samples retained a greater amount of undenatured type II collagen (> 50%) than those of the double heat processes. Undenatured type II collagen retention varied between single-heat processes and double-heat processing (67% and 18%, respectively; Table 4).

**Table 1:** Functional food and beverage prototype ingredients.

Product	Major Ingredients
Nutritional Bar	Soy protein nuggets (soy protein isolate, tapioca starch, salt), peanut butter fudge (corn syrup, invert sugar, peanut butter [peanuts, sugar, salt], sugar, palm kernel oil, peanut flour, milk protein isolate, soy lecithin, salt, vanilla extract, xanthan gum, carob seed gum, beta-carotene), chocolate layer (corn syrup, palm kernel oil, invert sugar, sugar, chocolate liquor, cocoa [processed with alkali], milk protein isolate, natural flavors, vitamin and mineral blend, UC-II® undenatured type II collagen
Brown Rice Chips	Corn masa flour, brown rice flour, tapioca starch, bumped rice**, s**alt, pepper, **f**lavors, UC-II® undenatured type II collagen, water
Chews	Gelatin, 250 bloom, maltitol syrup, red color FD&C 40, flavor, punch, FDC Yellow #5, flavor, orange, citric acid solution 50%, UC-II® undenatured type II collagen, water
Gummies	Tapioca syrup, cane sugar, gelatin, purified water, pectin, natural flavor, citric acid, sunflower oil, natural color, UC-II® undenatured type II collagen
Dairy Nutritional Beverage	Milk protein concentrate, 85%, soy protein isolate, canola oil, sodium phosphate, potassium citrate, table salt, lecithin, sugar, maltodextrin, tapioca, premix, vitamin/mineral 30% DV, gellan gum, cocoa, alkalized, sucralose, 25% solution, natural flavor, cocoa enhancer, natural flavor, vanilla 53 natural flavor, chocolate 51 sodium hydroxide, 20%, UC-II® undenatured type II collagen, water
Plant-Based Beverage	Pea protein, almond butter (smooth roasted), canola oil, sodium phosphate, potassium citrate, table salt, lecithin, sugar, maltodextrin, tapioca, premix, vitamin/mineral 30% DV, gellan gum, cocoa, alkalized, sucralose, 25% solution, natural flavor, cocoa enhancer, natural flavor, vanilla 53, natural flavor, chocolate 51 m sodium hydroxide, 20%, UC-II® undenatured type II collagen
Carbonated Juice Water	Grapefruit juice, orange juice, lemon juice, color, UC-II® undenatured type II collagen dispersion, water
Non-carbonated juice water	Flavor, lemon lime, sucrose, sodium citrate, dihydrate, sodium benzoate, UC-II® undenatured type II collagen dispersion, water

FD&C: Food, Drug, & Cosmetics; DV: daily value.

## 3 Experimental Design, Materials and Methods

The UC-II® collagen 40 mg providing 6% undenatured type II collagen (Lot #19FS106421), was provided by Lonza Capsules and Health Ingredients, Greenwood, SC. Bakery Barn, PA, and Mattson Co, CA, a contract food and beverage manufacturing company, procured the ingredients for this study from different suppliers (Table 1).

**Table 2:** Undenatured type II collagen recovery in food prototypes.

				Undenatured type II Collagen (mg)		
Product	Temp. (During the process;°C)	Time	pH	Pre-process	Post process	Undenatured type II collagen Retention (%)
Protein Food Bar (Baked)	121	20 min	NA	4.06	5.37	∼100
Salt + Pepper Brown Rice Chips	204 and 176	1–2 min	NA	2.4	1.59	66
BBQ Brown Rice Chips	204 and 176	1–2 min	NA	2.4	0.80	33
Sugar-Free Punch Chews	71 and 121	1–2 h	NA	2.4	2.36	98
Sugar-Free Orange Chews	71 and 121	1–2 h	NA	2.4	1.85	62
Gummies	71	30 min	4.7	1.32	1.26	96

min: minutes, h: hour; s: seconds; NA: pH data not collected.

**Table 3:** Undenatured type II collagen recovery in dairy beverage prototypes.

				Undenatured type II Collagen (mg)		
Product	Temperature (°C)	Time	pH	Pre-process	Post process	Undenatured type II collagen Retention (%)
Whole Milk	NA	NA	NA	2.4	1.53	64
Whole Milk (Heated)	37	10 min	NA	2.4	0.92	38
Dairy Nutritional (UHT)	143	6 s	7.0	2.4	1.94	81
Plant-based Nutritional (UHT)	143	6 s	7.0	2.4	1.4	59

min: minutes, h: hours; s: seconds; UHT: Ultra-high temperature; NA: pH data not collected.

**Table 4:** Undenatured type II collagen recovery in carbonated and non-carbonated beverages prototypes.

				Undenatured type II Collagen (mg)		
Product	Temperature (°C)	Time (sec)	pH	Pre-process	Post process	Undenatured type II collagen Retention (%)
Lemon-lime non-carbonated beverage (high-temperature–short-time (HTST) processing and hot filling)	88	30	3.0	2.4	0.78	33
Grapefruit carbonated juice water (single heat)	71–76	30	3.0–3.5	2.4	1.6	67
Grapefruit carbonated juice water (double heat)	88,21	30	7.0	2.4	0.44	18

min: minutes, h: hours; s: seconds; HTST: high-temperature–short-time.

For nutritional bar processing, all the listed wet and dry ingredients were mixed (Table 1). The ingredients were blended with UC-II® undenatured type II collagen (40 mg per bar) and passed through an extruder, which forms the mixture into the shape of a bar. The extruded material was then cut into the desired bar length and baked at 121 °C for 20 min and cooled at room temperature.

For the gummies processing, the gelatin was mixed with tapioca syrup, cane sugar, pectin, natural flavor (strawberry, orange), citric acid, natural color, and UC-II® undenatured type II collagen (40 mg). Then a purified water was added to form a slurry. The slurry was then heated to 71 °C for 30 min at a pH of 4.7. The slurry was moved to a storage tank via heated pipes to a hopper on the depositor. The time to deposit the slurry into molds was 30 min to 45 min and the molds were pre-treated with sunflower oil to prevent sticking. The slurry-filled molds were then passed through a chilling tunnel and rapidly cooled, thereby setting the mixture. After this, the gummies were removed from the molds and placed on trays to dry.

For the chews processing, 8.83% of gelatin 250 bloom (330 g) was mixed with water (15%) and stirred thoroughly. The gelatin slurry was placed into a 71°C water bath and allowed to be solubilized (for approximately 1 to 2 h). Separately, 0.46% of the citric acid solution, 0.05% Red Color FD&C 40, 0.63% flavor, punch, and UC-II® undenatured type II collagen 0.35% (40 mg) were mixed. The bulk sweetener, maltitol syrup (2800 g) was heated to 121 °C, and cooled to about 103 °C. The prepared gelatin slurry was added to the cooled maltitol syrup along with the acid, flavor and UC-II® mixture, mixed well, and warmed to maintain fluidity. The blended slurry solids were checked with a refractometer to measure the percent total dissolved solids (TDS) at about 78% to 80%. The prepared slurry was deposited into a prepared starch bed imprinted with the cavities of a desired shape and size of the finished piece and allowed to dry for 1 to 2 days. The samples were removed from dry starch and coated with a confectionary oil. The chews were individually wrapped and stored in an airtight container at room temperature for up to two weeks.

For the brown rice chip processing, the dry ingredients including corn masa flour, 21.63% (6.8 g), brown rice flour, 17.5% (5.5 g), tapioca starch, 8.27% (2.6 g), bumped rice, 7.95% (2.5 g), UC-II® undenatured type II collagen, 0.13% (40 mg) and a pinch of salt were blended. Water was heated to boiling temperature and set aside. The heated water was gradually added to the dry ingredients and mixed until a dough formed. The dough was covered and allowed to sit for 30 min. The dough was then rolled thin with a bakery sheeter to a width of approximately 1 to 1.5 mm, cut into desired shapes, and placed on a parchment-lined perforated baking sheet pan. The dough pieces were baked in a pre-heated oven at 204 °C for one minute. These pieces were fried in canola oil at 176 °C for 1.5 min to 2 min. Upon ceasing bubbling, the chips were removed, drained on a culinary paper-lined tray, and seasoned with salt and pepper.

For the dairy-based beverages, UC-II® undenatured type II collagen (40 mg) was added to 8 ounces of market-purchased whole milk and heated to 37 °C for 10 min. The mixture was then allowed to cool to room temperature.

The flavored dairy beverage was made by blending 85% milk protein concentrate, (187.3 g), soy protein isolate (0.65 g), canola oil (5.5 g), sodium phosphate (0.5 g), potassium citrate (0.5 g), table salt (0.1 g), lecithin (0.1 g), sugar (14 g), and maltodextrin tapioca (24.5 g). Water was heated to 49 °C, mixed with the blend, and sheared for 10 min. A cocoa slurry was prepared by adding hot water to alkalized cocoa (1.2 g). A slurry was then made from gellan gum and 25% sucralose solution (20 mg). The gum and sugar mixtures were combined under shear to the remaining ingredients (sugar and maltodextrin) were sheared into the mix. A vitamin and mineral premix (30% daily value) and UC-II® undenatured type II collagen (40 mg) were then added. Protein and cocoa slurries and other combined ingredients were added and mixed for 3 min, and the pH was adjusted to 7.0 with 20% sodium hydroxide. The mixture was processed through ultra-high temperature treatment (UHT) at 143 °C for 6 s by direct steam injection. The samples were homogenized at 2000/500 psi. The product was poured into bottles which were then refrigerated.

The plant-based beverage was prepared by blending pea protein (9 g), smooth roasted almond butter (8.5 g), canola oil (5.5 g), sodium phosphate (0.5 g), potassium citrate (0.5 g), table salt (0.1 g), lecithin (0.1 g), sugar (14 g), maltodextrin tapioca (24.5 g), and UC-II® undenatured type II collagen (40 mg). Water was heated to 49 °C, mixed with the blend, and sheared for 10 min. A cocoa slurry was prepared by adding hot water to alkalized cocoa (1.2 g). A slurry of gellan gum and 25% sucralose solution (0.02 g) was prepared.

For carbonated beverages using single heat processing conditions, carbonated grapefruit juice was prepared by stirring 88% water, 7% grapefruit juice, 2% orange juice, 0.8% lemon juice, color (0.0014%) and UC-II® dispersion until dissolved in a container. UC-II® dispersion in the formula was a slurry of 0.5682% of slurry (7.04 g) and delivered 40 mg UC-II® undenatured type II collagen into the water. The containers were rinsed with the remaining 20% water and blended with juice. The blend was transferred to a carbonator and bottled into 12-oz crown cap bottles. The sample was post-pasteurized in a 77 °C water bath, with a target of 71 °C to 76 °C. The beverages were kept cool in a water bath and refrigerated.

For double-heat processed carbonated beverages, carbonated grapefruit juice was prepared and added to the UC-II® dispersion. The containers were rinsed with the remaining 20% water and blended with juice. The blend was transferred to a high-temperature short-time process at 88 °C and held for 30 s. The sample was cooled to about 21 °C and transferred to a carbonator. The beverages were bottled into 12-oz crown cap bottles and kept cool in a water bath and then refrigerated afterwards.

For the agglomerated product (ready-to-mix powder), UC-II®-fortified agglomerated prototypes (with 2.5%, 5%, and 10% lecithin) were created. Water (140 mL) was added to a stainless steel beaker then heated to 38 °C. Lecithin 2.5%, 5%, and 10% was added to the water in respective beakers and agitated for 15 min. UC-II (40 mg) was added to the beaker. With a nozzle pressure of 0.50 bar and fluid bed air pressure of 35 bar, water was sprayed onto the fluid bed at 4 mL/min. The water temperature was between 33 °C and 36 °C, depending upon pump speed adjustment. The powder was heated to 41 °C, then cooled before being packaged.

The prototype samples and their suspended solids (in the case of beverage samples) were recovered by centrifugation at 15,700 rpm for 20 min and at a temperature of 40 °C; repeated spins were required to process the sample volume. The solids were recovered after centrifugation and dried by lyophilization. After extracting solids, an aliquot was weighed and subjected to the ELISA method to recover undenatured type II collagen using a multispecies Type II Collagen Detection Kit from Chondrex, Inc (Woodinville, WA, USA; Harris et al., 2021). The percent weight of undenatured type II collagen was calculated in the samples based on milligrams of undenatured type II collagen per grams of solid.

## CRediT authorship contribution statement

**George Pates:** Methodology, Writing – original draft. **Tyler White:** Supervision. **Shane Durkee:** Conceptualization, Validation, Writing – review & editing. **Zainulabedin Saiyed:** Conceptualization, Validation, Writing – review & editing.

## Declaration of Competing Interest

The authors declare the following financial interests/personal relationships which may be considered as potential competing interests: The authors are employees of Lonza Greenwood LLC., Greenwood, SC, USA.

## Data Availability

Tables and ELISA Raw Data (Original data) (Mendeley Data).
